# Aggregated 50-State, Regional, and State-Level Trends in State and Local Government Health Employees in the U.S. From 2000 Through 2023

**DOI:** 10.1016/j.focus.2025.100462

**Published:** 2025-10-30

**Authors:** Lijing Wei, Melody S. Goodman, Jemar R. Bather

**Affiliations:** Department of Biostatistics, NYU School of Global Public Health, New York, New York

**Keywords:** Change-point analysis, temporal trend, segmented regression, trend analysis, workforce development, health department

## Abstract

**Introduction:**

The authors investigated trends in U.S. state and local government health employees per million persons at the aggregated 50-state, regional, and state levels.

**Methods:**

The authors used repeated cross-sectional data from the Annual Survey of Public Employment & Payroll. Joinpoint regression was used to estimate average annual percentage changes and annual percentage changes from 2000 through 2023. State and local full-time and part-time government health employees included public health and several other categories of health workers.

**Results:**

State and local government health employees per million persons remained stable in the U.S. from 2000 through 2023. Heterogeneous trends in state and local government health employees were observed by region: Northeast (average annual percentage change=0.5% increase, 95% CI=0.3%, 0.7%), Midwest (average annual percentage change=0.9% increase, 95% CI=0.6%, 1.1%), South (average annual percentage change= −0.7% decrease, 95% CI= −0.9%, −0.5%), and West (average annual percentage change=0.1% increase, 95% CI= −0.1%, 0.2%). The authors observed further variation in state-stratified analyses.

**Conclusions:**

Most U.S. states experienced decreasing trends during the Great Recession (2007–2009) and increasing trends during the COVID-19 pandemic (2020–2023). Stable and long-term funding streams are essential to support consistent recruitment, training, and retention of state and local government health employees. Health policies should account for regional variations in health needs and employment trends when planning the state and local government health hiring.

## HIGHLIGHTS

• Joinpoint regression was used to examine U.S. government health employment trends.
• U.S. state and local government health employees remained stable from 2000 to 2023.
• State and local government health employment decreased in most states (2007–2009).
• State and local government health employment increased in many states (2020–2023).

## INTRODUCTION

State and local government health employees play a vital role in promoting health and preventing diseases, yet these governmental levels in the U.S. face persistent staffing shortages that threaten health employees’ ability to protect vulnerable populations.^1^ These decreases have persisted over time, with the federal, state, and local public health workforce declining from approximately 220 per 100,000 population in 1980 to 93 per 100,000 by 2014, representing a 58% erosion in staffing density.^2^ This challenge is acute for older adults, who require more intensive health services because of their increased susceptibility to infectious diseases and chronic conditions.^3^^,^^4^ The coronavirus disease 2019 (COVID-19) pandemic illustrated this vulnerability.^3^ Although the virus caused 83,000 related deaths from January 1, 2020, to May 18, 2020, in the U.S., older adults suffered disproportionately high mortality rates owing to underlying health conditions.^5^ Among 10,647 COVID-19 decedents with available supplementary data from February 12 to April 24 in 2020, 74.8% were aged ≥65 years.^5^ This crisis exposed critical gaps in state and local health infrastructure at a time when demographic trends intensified the challenge. The U.S. population aged ≥65 years is projected to grow from 58 million in 2022 to 82 million by 2050, with significant geographic variation in this distribution.^6^^,^^7^ In 2020, states such as Maine, Florida, West Virginia, and Vermont had older populations exceeding 20%, whereas states such as Utah, Alaska, Texas, and Georgia had proportions below 15%.^6^ Given these disparate demographic patterns and the growing demand for health services among aging populations, it is crucial to assess whether the supply of state and local government health employees can meet regional needs across different states.

Despite these staffing challenges, educational institutions have responded with significant growth in enrollment in public health degree programs.^8^, ^9^, ^10^ The number of undergraduate public health degrees awarded has surged by an average of 13.4% annually from 2001 to 2020, whereas graduate degrees quadrupled from 4,481 in 1992 to 19,124 in 2016.^8^^,^^9^ This expansion continued through 2018 across multiple institutions, suggesting a growth in public health graduates.^10^ However, this increase in graduates has not fully resolved state and local government staffing shortages, raising questions about retention, geographic distribution of graduates, and whether the pace of growth can match the rapidly increasing demands of an aging population.^11^, ^12^, ^13^, ^14^, ^15^

State and local government health staffing faces a retention crisis driven by systemic challenges, including low compensation and limited advancement opportunities.^12^, ^13^, ^14^, ^15^ Job satisfaction among state government public health staff declined across 16 states between 2014 and 2017.^12^ The potential consequences could be severe, with an increased rate (from 44% in 2014 to 48% in 2017) of state health agency staff planning to leave next year or retire within 5 years, whereas a third of state and local government public health employees nationwide actually quit between 2014 and 2017.^12^^,^^15^ People with several years of working experiences would be hard to replace with a newly trained recent graduate with no relevant experiences. Despite some recovery from an estimate of 206,500 state and local public health employment in 2019, reaching approximately 239,000 in 2022, staffing levels have returned to pre-2008 recession numbers, failing to account for population growth or the increasing complexity of health challenges since then.^16^ This stagnation means that the employee density remains far below historical levels, even as the need for health services continues to grow.

Although the trends mentioned earlier paint a concerning picture of the health employment capacity of state and local governments, a comprehensive analysis of long-term employment patterns and their implications remains limited.^8^, ^9^, ^10^^,^^12^, ^13^, ^14^, ^15^^,^^17^^,^^18^ Existing research has been fragmented, often focusing on specific subsets of health employees or conflating different employment sectors.^17^^,^^18^ For instance, 1 study tracking public health doctoral recipients from 2003 to 2015 showed declining the rates of nonacademic employment, but authors examined only a narrow segment of highly educated workers rather than the broader state and local government health employee population.^17^ Most employment trend analyses have focused on clinical healthcare providers in private practice rather than on government-employed health workers, predicting significant physician and nurse shortages without addressing the unique challenges facing state and local health agencies.^18^ This gap in research is significant given that state and local government health employees operate under different constraints than private sector providers, including civil service regulations, public funding limitations, and unique retention challenges.^19^ Without comprehensive data on trends in state and local government health employment trends across all education levels, geographic regions, and job classifications, policymakers cannot adequately address the staffing crisis threatening governmental health infrastructure. To address this critical research gap, the authors analyzed trends in state and local government health employment using data from the U.S. Census Bureau covering all 50 states from 2000 through 2023.

## METHODS

### Study Sample

The authors obtained U.S. state and local government health employment data, excluding data from federal agencies, for the years 2000 through 2023 from the Annual Survey of Public Employment & Payroll (ASPEP), a repeated cross-sectional survey collecting U.S. employment information on employees of 50 state government agencies and employees from up to 91,800 local governments, including the District of Columbia.^20^ In March of every year, ASPEP gathers data on the number of full-time, part-time, and full-time equivalent employees and their gross monthly income from most state governments through central payroll records.^20^ Data for the remaining state and local governments are recorded through online submissions.^20^ ASPEP classifies data into governmental functions using standardized item codes defined by the U.S. Census Bureau, such as health (032), financial administration (023), water supply (091), and public welfare (079).^20^ ASPEP’s recruitment criteria exclude employees from federal institutions.^20^ Additional information about ASPEP’s protocol has been provided elsewhere.^20^ Because the data were publicly available on the U.S. Census Bureau website, the New York University IRB considered this secondary data analysis exempt from review.^20^ This study followed the STROBE guidelines.^21^

### Measures

State and local government health employment data from ASPEP have been aggregated across a wide range of health-related occupations beyond public health, such as emergency medical, mental health, outpatient clinic, visiting nurses, substance use, food and sanitary inspection, and environmental health.^22^ Coroners and crime laboratory employees were excluded.^22^ Although ASPEP included the District of Columbia, it excluded Puerto Rico and other U.S. territories.^20^ To maintain consistent geographic representation, the authors excluded the District of Columbia in the analysis and focused on the 50 U.S. states. The annual total number of state and local government health employees per million persons was calculated as the number of state and local government health employees combined divided by the aggregated 50-state, census region, and state population estimates, obtained from the U.S. Census Bureau.^22^ The authors multiplied this value by 1,000,000 for interpretability. The Northeast region included Connecticut, Maine, Massachusetts, New Hampshire, New Jersey, New York, Pennsylvania, Rhode Island, and Vermont. The Midwest region included Illinois, Indiana, Iowa, Kansas, Michigan, Minnesota, Missouri, Nebraska, North Dakota, Ohio, South Dakota, and Wisconsin. The South region included Alabama, Arkansas, Delaware, Florida, Georgia, Kentucky, Louisiana, Maryland, Mississippi, North Carolina, Oklahoma, South Carolina, Tennessee, Texas, Virginia, and West Virginia. The West region included Alaska, Arizona, California, Colorado, Hawaii, Idaho, Montana, Nevada, New Mexico, Oregon, Utah, Washington, and Wyoming.^22^ The aggregated 50-state–level data included 50 states in the U.S. (excluding the District of Columbia, Puerto Rico, and other U.S. territories).^22^

### Statistical Analysis

Joinpoint regression models were used to examine trends of U.S. state and local full-time and part-time government health employees per million persons from 2000 through 2023. The average annual percentage change (AAPC) and annual percentage change (APC) were computed as measures of association with 95% CIs.^23^ The AAPC was used to explain the average rate of change in state and local government health employees per million persons from 2000 through 2023, and the APC was used to describe the rate of change within time frames identified by the joinpoint model. The significance level was set at 0.05 and was adjusted for multiple testing.^23^ Data cleaning was completed in R (Version 4.4.0) (R Core Team, R Foundation for Statistical Computing). All trend analyses were performed using Joinpoint Trend Analysis Software (Version 5.4.0.0).^24^ The authors characterized joinpoint trends using the National Cancer Institute’s categories: stable (−0.5≤APC≤0.5 and nonsignificant), nonsignificant change (APC< −0.5 or APC>0.5 and nonsignificant), rising (APC>0 and significant), and falling (APC<0 and significant).^25^

## RESULTS

**Figure 1:**
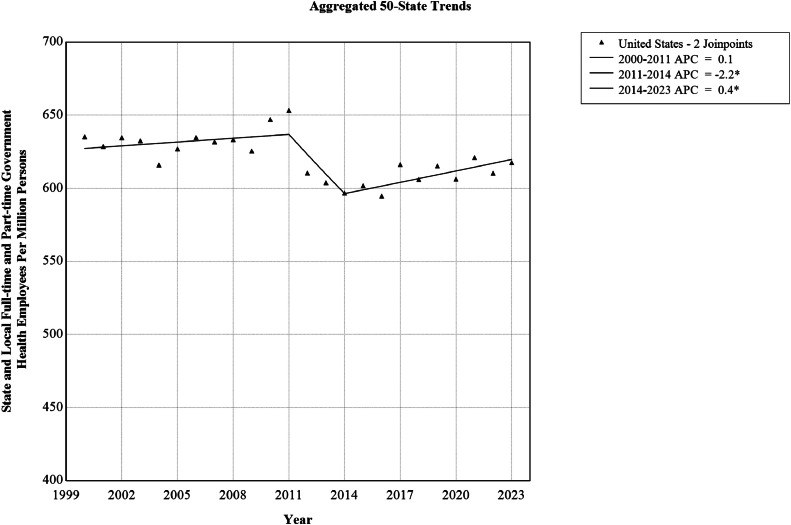
Aggregated 50-state trends in U.S. state and local full-time and part-time government health employees per million persons, 2000–2023. *Note*: The trend in U.S. state and local full-time and part-time government health employees per million persons was stable from 2000 through 2023. The asterisk (*) indicates that AAPC is statistically different from 0.0 at the 0.05 level. AAPC, average annual percentage change.

To examine aggregated 50-state trends, the annual total number of state and local full-time and part-time government health employees in the U.S. were calculated from 2000 through 2023 (Table 1). State and local government health employees per million persons decreased from 635.3 in 2000 to 617.7 in 2023, exhibiting a stable AAPC of −0.1% (95% CI= −0.12%, 0.03%) (Table 2). Joinpoint models indicated 3 U.S. trend segments (Table 2 and Figure 1), including a stable period from 2000 to 2011 (APC=0.14%, 95% CI= −0.02%, 0.39%), followed by a falling trend from 2011 to 2014 (APC= −2.2%, 95% CI= −2.7%, −1.0%) and then a rising trend from 2014 to 2023 (APC=0.4%, 95% CI=0.2%, 0.8%).

**Table 1:** State and Local Full-time and Part-time Government Health Employees Per Million Persons in the U.S., Using Data From the Annual Survey of Public Employment & Payroll, 2000–2023

Year	Full-time and part-time employees (*n*)	U.S. population (*n*)	State and local government health employees per million persons
2000	178,885	281,590,365	635.3
2001	178,770	284,394,451	628.6
2002	182,178	287,052,035	634.7
2003	183,126	289,539,431	632.5
2004	180,009	292,237,544	616.0
2005	184,934	294,949,463	627.0
2006	189,054	297,809,231	634.8
2007	189,954	300,656,803	631.8
2008	192,212	303,513,730	633.3
2009	191,526	306,179,301	625.5
2010	199,780	308,716,440	647.1
2011	203,159	310,937,074	653.4
2012	191,225	313,196,066	610.6
2013	190,439	315,343,134	603.9
2014	189,576	317,638,680	596.8
2015	192,565	319,959,763	601.8
2016	191,677	322,255,496	594.8
2017	199,854	324,290,633	616.3
2018	197,591	325,985,954	606.1
2019	201,551	327,533,774	615.4
2020	200,629	330,856,094	606.4
2021	205,829	331,379,940	621.1
2022	203,031	332,600,462	610.4
2023	206,452	334,235,923	617.7

**Table 2:** Aggregated 50-State, Regional, and State-Level Trends in U.S. State and local Full-Time and Part-Time Government Health Employees Per Million Persons, Using Data From the Annual Survey of Public Employment & Payroll, 2000–2023

Region	Trend segment	Segment endpoints		APC (95% CI)	AAPC (95% CI)
U.S.	1	2000	2011	0.1 (−0.0, 0.4)	−0.1 (−0.1, 0.0)
	2	2011	2014	−2.2 (−2.7, −1.0)*	
	3	2014	2023	0.4 (0.2, 0.8)*	
West	1	2000	2010	−1.1 (−1.8, −0.6)*	0.1 (−0.1, 0.2)
	2	2010	2023	1.0 (0.6, 1.4)*	
Alaska	1	2000	2018	−0.9 (−2.4, −0.3)*	0.3 (−0.5, 0.9)
	2	2018	2023	4.6 (0.7, 14.8)*	
Arizona	1	2000	2009	0.5 (−0.8, 3.6)	−0.9 (−1.3, −0.3)*
	2	2009	2016	−5.2 (−10.8, −3.3)*	
	3	2016	2023	1.8 (0.1, 5.2)*	
California	1	2000	2005	−2.2 (−6.3, −0.2)*	0.3 (−0.1, 0.6)
	2	2005	2017	1.8 (1.3, 4.9)*	
	3	2017	2023	−0.6 (−5.1, 0.7)	
Colorado	1	2000	2007	−0.7 (−2.0, −0.3)*	1.1 (1.0, 1.2)*
	2	2007	2010	0.9 (−0.3, 1.6)	
	3	2010	2014	−1.6 (−2.8, −0.6)*	
	4	2014	2020	1.5 (0.9, 2.4)*	
	5	2020	2023	8.8 (7.4, 11.2)*	
Hawaii	1	2000	2008	−1.6 (−3.3, −0.3)*	−1.4 (−1.9, −1.2)*
	2	2008	2011	−8.9 (−10.1, −0.2)*	
	3	2011	2021	−0.3 (−9.1, 0.4)	
	4	2021	2023	5.6 (0.1, 8.7)*	
Idaho	1	2000	2010	−2.4 (−4.4, −1.0)*	0.4 (−0.2, 0.8)
	2	2010	2013	17.7 (7.7, 21.9)*	
	3	2013	2023	−1.7 (−3.6, −0.4)*	
Montana	1	2000	2005	−3.1 (−8.3, −1.0)*	−2.2 (−2.5, −1.9)*
	2	2005	2012	2.2 (1.0, 6.0)*	
	3	2012	2023	−4.5 (−5.2, −3.9)*	
Nevada	1	2000	2023	0.1 (−0.2, 0.5)	0.1 (−0.2, 0.5)
New Mexico	1	2000	2023	−1.4 (−1.8, −1.0)*	−1.4 (−1.8, −1.0)*
Oregon	1	2000	2005	2.1 (0.0, 5.5)*	−1.4 (−1.9, −0.9)*
	2	2005	2008	−22.7 (−25.0, −17.3)*	
	3	2008	2015	7.3 (5.7, 10.0)*	
	4	2015	2019	−8.4 (−13.1, −4.6)*	
	5	2019	2023	5.2 (1.6, 12.4)*	
Utah	1	2000	2006	−0.5 (−1.5, 1.6)	−0.2 (−0.4, 0.1)
	2	2006	2009	−7.0 (−8.5, −3.8)*	
	3	2009	2016	−0.4 (−2.0, 1.3)	
	4	2016	2023	3.3 (2.3, 6.0)*	
Washington	1	2000	2011	−1.1 (−2.3, −0.2)*	1.0 (0.7, 1.4)*
	2	2011	2023	3.0 (2.2, 4.2)*	
Wyoming	1	2000	2013	2.9 (2.3, 3.5)*	0.9 (0.6, 1.2)*
	2	2013	2023	−1.6 (−2.6, −0.8)*	
South	1	2000	2011	0.3 (−0.1, 0.9)	−0.7 (−0.9, −0.5)*
	2	2011	2014	−5.6 (−6.8, −2.7)*	
	3	2014	2023	−0.3 (−0.9, 0.8)	
Alabama	1	2000	2011	1.2 (0.8, 1.7)*	0.5 (0.2, 0.7)*
	2	2011	2015	−9.7 (−12.2, −7.6)*	
	3	2015	2018	15.6 (11.7, 17.9)*	
	4	2018	2023	−1.1 (−3.1, 0.1)	
Arkansas	1	2000	2008	−0.3 (−1.2, 0.9)	−2.6 (−2.9, −2.3)*
	2	2008	2018	−4.8 (−7.0, −4.2)*	
	3	2018	2023	−1.7 (−3.4, 2.4)	
Delaware	1	2000	2008	0.7 (−0.5, 4.7)	−0.8 (−1.2, −0.4)*
	2	2008	2023	−1.6 (−3.1, −1.1)*	
Florida	1	2000	2007	1.4 (0.2, 3.0)*	−1.7 (−2.0, −1.3)*
	2	2007	2016	−5.3 (−7.0, −4.4)*	
	3	2016	2023	0.1 (−1.2, 2.1)	
Georgia	1	2000	2008	1.7 (−0.2, 5.2)	−0.9 (−1.4, −0.2)*
	2	2008	2015	−6.7 (−14.1, −4.4)*	
	3	2015	2023	1.9 (−0.1, 6.0)	
Kentucky	1	2000	2008	0.0 (−1.1, 0.8)	2.0 (1.8, 2.3)*
	2	2008	2011	10.5 (6.5, 12.2)*	
	3	2011	2023	1.4 (0.8, 1.8)*	
Louisiana	1	2000	2003	−11.2 (−19.4, −5.5)*	−1.6 (−2.1, −1.0)*
	2	2003	2009	1.6 (−0.1, 7.6)	
	3	2009	2012	−16.4 (−19.7, −11.3)*	
	4	2012	2015	13.1 (7.1, 17.1)*	
	5	2015	2023	0.9 (−1.3, 2.0)	
Maryland	1	2000	2007	−0.0 (−1.0, 2.9)	0.1 (−0.3, 0.4)
	2	2007	2015	−2.8 (−5.9, −2.1)*	
	3	2015	2018	12.6 (7.4, 15.3)*	
	4	2018	2023	−2.2 (−4.9, −0.7)*	
Mississippi	1	2000	2008	−1.1 (−3.9, −0.0)*	−0.9 (−1.2, −0.6)*
	2	2008	2014	2.4 (0.7, 5.8)*	
	3	2014	2018	−5.6 (−8.3, −3.0)*	
	4	2018	2023	−0.5 (−2.4, 4.1)	
North Carolina	1	2000	2008	6.1 (4.5, 8.3)*	−3.2 (−3.7, −2.8)*
	2	2008	2011	−21.8 (−24.3, −14.2)*	
	3	2011	2023	−4.0 (−4.9, −2.9)*	
Oklahoma	1	2000	2003	17.7 (11.5, 26.0)*	0.9 (0.5, 1.4)*
	2	2003	2019	−2.0 (−3.7, −1.6)*	
	3	2019	2023	1.4 (−1.4, 7.5)	
South Carolina	1	2000	2009	−4.4 (−6.2, −3.8)*	−3.0 (−3.3, −2.8)*
	2	2009	2018	−2.8 (−3.6, −1.7)*	
	3	2018	2021	2.6 (−2.3, 4.0)	
	4	2021	2023	−6.0 (−9.3, −0.9)*	
Tennessee	1	2000	2010	5.5 (3.1, 9.9)*	0.4 (−0.5, 1.2)
	2	2010	2023	−3.3 (−5.8, −1.8)*	
Texas	1	2000	2008	−1.9 (−7.6, 0.5)	−0.4 (−1.4, 0.3)
	2	2008	2011	18.7 (5.7, 25.3)*	
	3	2011	2023	−3.7 (−6.2, −2.1)*	
Virginia	1	2000	2013	−0.5 (−0.9, −0.2)*	0.1 (−0.1, 0.3)
	2	2013	2016	8.0 (4.2, 9.5)*	
	3	2016	2023	−2.0 (−3.3, −1.2)*	
West Virginia	1	2000	2005	3.7 (1.7, 10.2)*	1.4 (1.0, 2.0)*
	2	2005	2008	−4.3 (−7.1, −0.5)*	
	3	2008	2016	1.2 (0.4, 6.3)*	
	4	2016	2019	−4.1 (−7.4, −0.5)*	
	5	2019	2023	7.8 (4.5, 16.6)*	
Midwest	1	2000	2010	−1.0 (−1.6, −0.4)*	0.9 (0.6, 1.1)*
	2	2010	2014	6.5 (3.8, 9.4)*	
	3	2014	2020	−1.1 (−3.8, 0.2)	
	4	2020	2023	3.8 (0.6, 8.4)*	
Illinois	1	2000	2018	−2.3 (−2.6, −2.1)*	−1.6 (−1.8, −1.4)*
	2	2018	2023	0.9 (−0.6, 3.9)	
Indiana	1	2000	2008	1.1 (0.5, 1.8)*	−0.4 (−0.5, −0.2)*
	2	2008	2012	−4.1 (−5.9, −2.5)*	
	3	2012	2023	−0.1 (−0.4, 0.4)	
Iowa	1	2000	2008	2.7 (1.3, 5.4)*	0.2 (−0.4, 0.8)
	2	2008	2018	−1.3 (−5.3, −0.5)*	
	3	2018	2021	12.0 (6.1, 16.3)*	
	4	2021	2023	−16.8 (−23.5, −7.6)*	
Kansas	1	2000	2009	2.4 (1.1, 4.5)*	1.7 (1.3, 2.3)*
	2	2009	2015	−4.3 (−10.8, −1.8)*	
	3	2015	2023	5.8 (3.9, 9.1)*	
Michigan	1	2000	2010	−0.1 (−2.5, 1.7)	6.1 (5.3, 6.7)*
	2	2010	2013	44.6 (26.8, 51.7)*	
	3	2013	2023	2.6 (0.1, 4.4)*	
Minnesota	1	2000	2010	0.5 (−0.6, 1.3)	0.9 (0.3, 1.4)*
	2	2010	2013	26.8 (18.2, 30.0)*	
	3	2013	2018	0.4 (−2.7, 3.6)	
	4	2018	2021	−20.8 (−23.6, −16.2)*	
	5	2021	2023	6.1 (−3.0, 14.6)	
Missouri	1	2000	2009	−2.9 (−5.0, −2.3)*	−1.2 (−1.5, −0.9)*
	2	2009	2021	−1.1 (−1.8, −0.4)*	
	3	2021	2023	6.4 (0.7, 9.2)*	
Nebraska	1	2000	2015	−2.7 (−3.2, −0.5)*	−1.4 (−1.9, −0.8)*
	2	2015	2019	−7.5 (−12.1, −3.8)*	
	3	2019	2023	10.4 (5.8, 19.6)*	
North Dakota	1	2000	2023	0.8 (0.2, 1.4)*	0.8 (0.2, 1.4)*
Ohio	1	2000	2008	−0.2 (−1.5, 4.4)	1.1 (0.7, 1.6)*
	2	2008	2019	−3.3 (−5.5, −2.6)*	
	3	2019	2023	17.2 (12.0, 22.2)*	
South Dakota	1	2000	2005	4.5 (0.7, 14.5)*	−0.4 (−1.0, 0.1)
	2	2005	2023	−1.7 (−2.7, −1.2)*	
Wisconsin	1	2000	2010	−3.8 (−5.0, −3.0)*	−1.1 (−1.4, −0.8)*
	2	2010	2023	1.0 (0.4, 1.8)*	
Northeast	1	2000	2005	0.1 (−2.5, 1.2)	0.5 (0.3, 0.7)*
	2	2005	2009	2.4 (−2.0, 4.1)	
	3	2009	2015	−2.4 (−4.2, −0.1)*	
	4	2015	2018	5.2 (2.0, 6.6)*	
	5	2018	2023	0.4 (−1.5, 1.3)	
Connecticut	1	2000	2007	−3.0 (−6.6, −0.4)*	3.4 (2.6, 4.3)*
	2	2007	2010	37.2 (20.9, 44.6)*	
	3	2010	2018	−4.2 (−11.2, −2.3)*	
	4	2018	2023	8.1 (2.8, 19.8)*	
Maine	1	2000	2003	3.6 (0.5, 8.9)*	−1.2 (−1.5, −0.9)*
	2	2003	2015	−3.2 (−4.2, −2.7)*	
	3	2015	2023	−0.0 (−0.9, 1.7)	
Massachusetts	1	2000	2016	−1.5 (−2.1, −1.0)*	0.3 (−0.1, 0.6)
	2	2016	2023	4.3 (2.6, 7.4)*	
New Hampshire	1	2000	2002	7.6 (4.2, 11.3)*	−0.9 (−1.1, −0.7)*
	2	2002	2005	−7.7 (−9.3, −5.9)*	
	3	2005	2008	5.5 (3.0, 7.1)*	
	4	2008	2014	−4.4 (−6.1, −3.6)*	
	5	2014	2023	−0.1 (−0.6, 0.6)	
New Jersey	1	2000	2002	−7.3 (−13.5, 2.5)	0.7 (0.3, 1.3)*
	2	2002	2006	17.0 (11.8, 24.2)*	
	3	2006	2023	−1.8 (−2.3, −1.3)*	
New York	1	2000	2005	−0.9 (−3.9, 0.5)	−0.7 (−1.0, −0.5)*
	2	2005	2009	2.2 (−5.4, 4.3)	
	3	2009	2014	−5.8 (−8.3, 4.4)	
	4	2014	2017	5.1 (−1.2, 6.8)	
	5	2017	2023	−1.0 (−2.7, 0.1)	
Pennsylvania	1	2000	2011	3.5 (2.6, 5.3)*	4.5 (4.1, 5.1)*
	2	2011	2015	−3.7 (−8.2, 0.0)	
	3	2015	2018	44.9 (33.5, 51.4)*	
	4	2018	2023	−6.2 (−10.2, −3.5)*	
Rhode Island	1	2000	2008	−2.1 (−2.7, −1.2)*	−1.7 (−1.8, −1.5)*
	2	2008	2012	−6.7 (−8.9, −4.9)*	
	3	2012	2023	0.5 (0.1, 1.0)*	
Vermont	1	2000	2007	6.2 (4.0, 9.2)*	−0.3 (−0.7, 0.2)
	2	2007	2011	−14.7 (−20.3, −10.0)*	
	3	2011	2023	1.2 (0.2, 2.5)*	

*Note*: Asterisk (*) indicates that AAPC/APC is statistically different from 0.0 at the 0.05 level.

AAPC, average annual percentage change; APC, annual percentage change.

**Figure 2:**
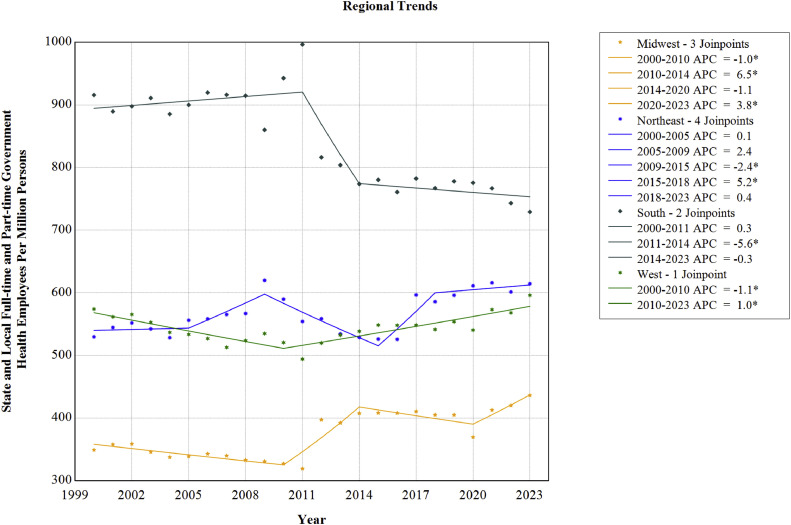
Regional trends in U.S. state and local full-time and part-time government health employees per million persons, 2000–2023. *Note*: Trends in U.S. state and local full-time and part-time government health employees per million persons varied across different regions from 2000 through 2023. The asterisk (*) indicates that AAPC is statistically different from 0.0 at the 0.05 level. AAPC, average annual percentage change.

As indicated in Table 2 and Figure 2, the authors showed differential overall trends by census region from 2000 through 2023. The trend was stable in the West (AAPC=0.1%, 95% CI= −0.1%, 0.2%). However, the Midwest (AAPC=0.9%, 95% CI=0.6%, 1.1%) and Northeast (AAPC=0.5%, 95% CI=0.3%, 0.7%) regions demonstrated significant rising trends, and the South region (AAPC= −0.7%, 95% CI= −0.9%, −0.5%) showed a falling trend from 2000 through 2023.

Joinpoint analyses generated 5 trend segments for the Northeast region, compared with 4 for the Midwest region, 3 for the South region, and 2 for the West region (Table 2). This suggests that the Northeast region demonstrated considerable volatility in state and local government health employees per million persons over time compared with other census regions. Notably, the Northeast experienced a rising trend from 2015 to 2018 (APC=5.2%, 95% CI=2.0%, 6.6%), followed by stability from 2018 to 2023 (APC=0.4%, 95% CI= −1.5%, 1.3%). The Midwest region experienced substantial growth from 2010 to 2014 (APC=6.5%, 95% CI=3.8%, 9.4%) and from 2020 to 2023 (APC=3.8%, 95% CI=0.6%, 8.4%). Conversely, a period of significant decline was exhibited in the South region from 2011 to 2014 (APC= −5.6%, 95% CI= −6.8%, −2.7%), followed by a nonsignificant change from 2014 to 2023 (APC= −0.3%, 95% CI= −0.9%, 0.8%). The West region showed a significant decrease from 2000 to 2010 (APC= −1.1%, 95% CI= −1.8%, −0.6%) and a significant increase from 2010 to 2023 (APC=1.0%, 95% CI=0.6%, 1.4%).

Most U.S. states (*n*=27) experienced changes in the trend of state and local government health employees per million persons between 2007 and 2009. Among these 27 states, 18 states across different regions experienced significant decreases between 2007 and 2009, including Arizona in the West (APC= −5.2%, 95% CI= −10.8%, −3.3%), North Carolina in the South (APC= −21.8%, 95% CI= −24.3%, −14.2%), Indiana in the Midwest (APC= −4.1%, 95% CI= −5.9%, −2.5%), and Vermont in the Northeast (APC= −14.7%, 95% CI= −20.3%, −10.0%). In addition, 6 states from the West, South, and Northeast regions experienced significant increases during this period, including Oregon in the West (APC=7.3%, 95% CI=5.7%, 10.0%), Texas in the South (APC=18.7%, 95% CI=5.7%, 25.3%), and Connecticut in the Northeast (APC=37.2%, 95% CI=20.9%, 44.6%).

In addition to the significant changes between 2007 and 2009, some significant increasing changes were also observed between 2020 and 2023, a time frame overlapping with the COVID-19 pandemic. Within the West region, the authors observed a significant increase in Colorado from 2020 to 2023 (APC=8.8%, 95% CI=7.4%, 11.2%) and in Hawaii from 2021 to 2023 (APC=5.6%, 95% CI=0.1%, 8.7%). A similar increasing trend was observed in Missouri in the Midwest region from 2021 to 2023 (APC=6.4%, 95% CI=0.7%, 9.2%).

These results remained robust in the sensitivity analysis of full-time employees, excluding part-time state and local government health employees from the analyses (Appendix Table 1, available online). For example, 29 states experienced changes in the trend of full-time government health employees per million persons between 2007 and 2009, and several states experienced a significant increase during the COVID-19 pandemic (2020–2023) (Appendix Table 1, available online), including West Virginia in the South region from 2021 to 2023 (APC=8.0%, 95% CI=0.6%, 11.5%), Minnesota in the Midwest region from 2021 to 2023 (APC=4.6%, 95% CI=0.7%, 8.0%), and Vermont in the Northeast region from 2020 to 2023 (APC=6.9%, 95% CI=4.2%, 11.8%).

## DISCUSSION

Using data from the ASPEP and the U.S. Census Bureau, the authors examined trends in state and local government health employees from 2000 through 2023. This analysis showed complex patterns that varied significantly by time period and geography. At the aggregated 50-state level, the density (per million population) of state and local government health employees remained stable over the 24-year study period, masking temporal and geographic variations. Most notably, employment declined from 2011 to 2014, likely reflecting budget cuts to the Health Resources and Services Administration and other health-related agencies that support local and state health departments through programs.^26^ This pattern aligns with extensive research demonstrating public health workforce losses during and after the Great Recession.^27^, ^28^, ^29^, ^30^ Previous studies showed that local health departments lost over 23,000 positions from 2008 to 2009, with 91% of territorial health agencies experiencing job losses through layoffs or attrition.^28^^,^^29^ These cuts resulted from severe budgetary shortfalls as state and local revenues plummeted during the economic downturn.^30^ In addition, the observed declines may partly reflect efficiency gains from technology and other factors.

The COVID-19 pandemic marked a dramatic reversal of this trend. Many states experienced increasing employment from 2020 through 2023, reflecting emergency hiring efforts to address the crisis.^1^ However, this reactive expansion highlighted the risks of chronic underinvestment in public health infrastructure.^1^ The pandemic exposed critical vulnerabilities in emergency preparedness, coordination systems, and surge capacity, which had been weakened by years of budget constraints.^31^

Geographic variations in employment trends suggest that one-size-fits-all workforce policies may be inadequate. Census regions showed divergent patterns that may reflect differences in state fiscal capacity, political priorities, and population health needs. Previous research indicates that rural health departments provide more direct services while engaging in fewer population-focused activities, which could partially explain regional workforce variations.^32^ These disparities underscore the need for tailored workforce strategies that account for regional contexts and constraints.

### Limitations

This study has several limitations. First, the ASPEP data collection occurs annually in March, which may not capture year-end employment levels used by many state systems.^20^ Second, the employment category combines various health occupations, including public health workers, visiting nurses, and other health-adjacent personnel, preventing analysis of specific workforce sectors.^20^ This aggregation may obscure important trends within different occupational categories. Third, the U.S. Census Bureau imputes missing data for nonresponding units, potentially introducing bias.^20^ Fourth, given that the unique needs of each state and local department are changing over time and that the implementation of tools, such as artificial intelligence, could impact appropriate staffing levels, the correct employee density is unknown. Finally, the analysis excludes Puerto Rico and other U.S. territories, limiting generalizability beyond the mainland U.S.^33^

Despite these limitations, the findings provide the most comprehensive analysis to date of health employment trends in state and local government. The data demonstrate that the density of state and local government health employees has experienced significant shocks from economic downturns and public health emergencies, with recovery patterns varying substantially across states and regions. Future research should develop more granular data systems capable of tracking specific public health occupations and including U.S. territories. Such improvements would enable more targeted workforce planning and better preparation for future challenges.

## CONCLUSIONS

This study documents state and local government health employment rates marked by vulnerability to economic shocks and reactive rather than proactive staffing patterns. Although national employment density remained relatively stable from 2000 to 2023, this masks significant volatility (e.g., widespread staffing cuts during the Great Recession followed by emergency hiring during COVID-19). These fluctuations demonstrate that current workforce planning fails to maintain the consistent capacity needed for effective public health protection. The pandemic-era employment gains face immediate jeopardy. The American Rescue Plan Act funding has already expired, and the Public Health Infrastructure Grant program ends in 2027, threatening to reverse recent workforce expansion.^34^^,^^35^ Without sustainable funding mechanisms, state and local health agencies risk returning to prepandemic staffing inadequacies just as demographic pressures intensify.

The findings underscore 3 critical policy priorities. First, health agencies need stable, long-term funding streams that transcend crisis cycles to support consistent recruitment, training, and retention efforts.^16^ Second, workforce planning must account for substantial regional variations in health needs and employment trends, moving beyond 1-size-fits-all approaches. Third, agencies should investigate whether pandemic-era innovations such as hybrid work arrangements can improve retention in a traditionally challenging employment sector.^36^, ^37^, ^38^

Future research should track postpandemic employment trends to assess whether recent gains prove sustainable, evaluate the effectiveness of different retention strategies across regions, and develop more granular data systems to distinguish between various health occupations.^39^ Only through sustained investment and evidence-based workforce planning can state and local governments build the resilient health infrastructure necessary to protect an aging population and respond to future public health challenges.

## CRediT authorship contribution statement

**Lijing Wei:** Conceptualization, Methodology, Software, Formal analysis, Investigation, Writing – original draft, Visualization. **Melody S. Goodman:** Investigation, Writing – review & editing. **Jemar R. Bather:** Conceptualization, Methodology, Investigation, Writing – review & editing, Project administration.

## Appendix Supplementary materials

Image, application 1

## Footnotes

Supplementary material associated with this article can be found in the online version at doi:10.1016/j.focus.2025.100462.
