# Supplementary material for: Aggregated 50-State, Regional, and State-Level Trends in State and Local Government Health Employees in the U.S. From 2000 Through 2023
Source: AJPM Focus. 2025 Oct 30;5(2):100462. doi: 10.1016/j.focus.2025.100462 (PMC12934315; doi:10.1016/j.focus.2025.100462)
Supplement: Supplementary file 1 [file mmc1.docx]

**Appendix Table 1.** Aggregated 50-State, Regional, and State-Level Trends in U.S. State and Local Full-Time Government Health Employees Per Million Persons, Using Data from the Annual Survey of Public Employment & Payroll, 2000-2023.

| **Region** | **Trend Segment** | **Segment Endpoints** | | **APC (95% CI)** | **AAPC (95% CI)** |
| --- | --- | --- | --- | --- | --- |
| **United States** | 1 | 2000 | 2011 | 0.2 (-0.0, 0.4) | -0.0 (-0.1, 0.1) |
|  | 2 | 2011 | 2014 | -2.7 (-3.3, -1.3)* |  |
|  | 3 | 2014 | 2023 | 0.6 (0.4, 1.0)* |  |
| **West** | 1 | 2000 | 2006 | -1.6 (-3.9, 0.2) | 0.3 (0.1, 0.5)* |
|  | 2 | 2006 | 2012 | -0.0 (-1.6, 2.5) |  |
|  | 3 | 2012 | 2023 | 1.5 (-0.5, 3.0) |  |
| Alaska | 1 | 2000 | 2023 | -0.1 (-0.6, 0.3) | -0.1 (-0.6, 0.3) |
| Arizona | 1 | 2000 | 2009 | 0.4 (-0.9, 3.3) | -0.9 (-1.3, -0.4)* |
|  | 2 | 2009 | 2016 | -4.9 (-10.2, -3.2)* |  |
|  | 3 | 2016 | 2023 | 1.7 (-0.0, 4.7) |  |
| California | 1 | 2000 | 2005 | -1.5 (-6.4, 1.0) | 0.9 (0.6, 1.4)* |
|  | 2 | 2005 | 2023 | 1.6 (1.1, 3.5)* |  |
| Colorado | 1 | 2000 | 2007 | -0.6 (-2.3, -0.1)* | 1.4 (1.3, 1.6)* |
|  | 2 | 2007 | 2010 | 1.5 (0.1, 2.3)* |  |
|  | 3 | 2010 | 2014 | -1.6 (-3.0, -0.5)* |  |
|  | 4 | 2014 | 2020 | 1.8 (1.1, 3.2)* |  |
|  | 5 | 2020 | 2023 | 10.0 (8.4, 12.9)* |  |
| Hawaii | 1 | 2000 | 2002 | -6.0 (-8.5, -1.6)* | -1.6 (-1.9, -1.3)* |
|  | 2 | 2002 | 2008 | -0.9 (-8.7, 1.6) |  |
|  | 3 | 2008 | 2011 | -9.2 (-10.4, 0.9) |  |
|  | 4 | 2011 | 2021 | -0.1 (-1.9, 0.4) |  |
|  | 5 | 2021 | 2023 | 5.7 (1.0, 8.3)* |  |
| Idaho | 1 | 2000 | 2010 | -1.8 (-3.6, -0.6)* | 0.6 (0.1, 1.0)* |
|  | 2 | 2010 | 2013 | 15.8 (7.0, 19.5)* |  |
|  | 3 | 2013 | 2023 | -1.2 (-2.9, -0.1)* |  |
| Montana | 1 | 2000 | 2005 | -2.7 (-7.8, 0.9) | -2.0 (-2.4, -1.6)* |
|  | 2 | 2005 | 2009 | 2.8 (-3.9, 6.1) |  |
|  | 3 | 2009 | 2012 | -4.2 (-6.8, 3.4) |  |
|  | 4 | 2012 | 2015 | 1.3 (-7.4, 3.8) |  |
|  | 5 | 2015 | 2023 | -4.3 (-8.1, -0.5)* |  |
| Nevada | 1 | 2000 | 2023 | 0.3 (-0.0, 0.6) | 0.3 (-0.0, 0.6) |
| New Mexico | 1 | 2000 | 2023 | -1.1 (-1.5, -0.7)* | -1.1 (-1.5, -0.7)* |
| Oregon | 1 | 2000 | 2005 | 2.1 (-0.1, 5.4) | -1.0 (-1.4, -0.5)* |
|  | 2 | 2005 | 2008 | -22.9 (-25.2, -17.6)* |  |
|  | 3 | 2008 | 2015 | 8.4 (6.8, 11.0)* |  |
|  | 4 | 2015 | 2019 | -8.0 (-12.8, -4.3)* |  |
|  | 5 | 2019 | 2023 | 5.5 (2.0, 12.1)* |  |
| Utah | 1 | 2000 | 2006 | -0.8 (-1.5, 0.7) | -0.2 (-0.4, 0.0) |
|  | 2 | 2006 | 2009 | -6.2 (-7.4, -3.8)* |  |
|  | 3 | 2009 | 2015 | -0.6 (-1.9, 1.1) |  |
|  | 4 | 2015 | 2023 | 2.9 (2.2, 4.1)* |  |
| Washington | 1 | 2000 | 2010 | -1.8 (-3.1, -0.8)* | 1.5 (1.1, 1.9)* |
|  | 2 | 2010 | 2023 | 4.1 (3.3, 5.0)* |  |
| Wyoming | 1 | 2000 | 2013 | 3.8 (3.1, 4.6)* | 1.3 (1.0, 1.7)* |
|  | 2 | 2013 | 2023 | -1.8 (-3.0, -0.7)* |  |
| **South** | 1 | 2000 | 2011 | 0.4 (-0.0, 1.0) | -0.8 (-1.0, -0.6)* |
|  | 2 | 2011 | 2014 | -5.7 (-6.8, -2.8)* |  |
|  | 3 | 2014 | 2023 | -0.6 (-1.1, 0.6) |  |
| Alabama | 1 | 2000 | 2012 | 1.0 (0.6, 1.5)* | 0.5 (0.2, 0.7)* |
|  | 2 | 2012 | 2015 | -12.7 (-14.5, -9.1)* |  |
|  | 3 | 2015 | 2018 | 16.2 (11.9, 19.1)* |  |
|  | 4 | 2018 | 2023 | -0.9 (-3.2, 0.3) |  |
| Arkansas | 1 | 2000 | 2008 | -0.0 (-1.4, 1.6) | -2.5 (-2.9, -2.1)* |
|  | 2 | 2008 | 2018 | -4.9 (-8.2, -3.6)* |  |
|  | 3 | 2018 | 2023 | -1.6 (-3.8, 3.5) |  |
| Delaware | 1 | 2000 | 2008 | 1.4 (0.2, 4.5)* | -0.6 (-1.0, -0.2)* |
|  | 2 | 2008 | 2023 | -1.7 (-2.5, -1.2)* |  |
| Florida | 1 | 2000 | 2004 | -0.7 (-4.4, 1.3) | -2.1 (-2.4, -1.8)* |
|  | 2 | 2004 | 2007 | 3.2 (-6.9, 4.8) |  |
|  | 3 | 2007 | 2017 | -5.4 (-7.1, -4.0)* |  |
|  | 4 | 2017 | 2021 | 2.7 (-3.2, 5.7) |  |
|  | 5 | 2021 | 2023 | -5.0 (-8.9, -0.1)* |  |
| Georgia | 1 | 2000 | 2007 | 2.5 (1.1, 4.6)* | -1.3 (-1.7, -1.0)* |
|  | 2 | 2007 | 2014 | -5.8 (-9.9, -4.3)* |  |
|  | 3 | 2014 | 2023 | -0.8 (-1.8, 1.1) |  |
| Kentucky | 1 | 2000 | 2008 | -0.6 (-1.9, 0.4) | 2.0 (1.7, 2.3)* |
|  | 2 | 2008 | 2011 | 12.1 (7.2, 14.2)* |  |
|  | 3 | 2011 | 2023 | 1.4 (0.7, 1.9)* |  |
| Louisiana | 1 | 2000 | 2003 | -10.8 (-18.6, -5.8)* | -1.6 (-2.1, -1.1)* |
|  | 2 | 2003 | 2009 | 1.8 (0.2, 7.0)* |  |
|  | 3 | 2009 | 2012 | -16.3 (-19.1, -11.6)* |  |
|  | 4 | 2012 | 2015 | 12.5 (7.2, 15.9)* |  |
|  | 5 | 2015 | 2023 | 0.5 (-1.3, 1.5) |  |
| Maryland | 1 | 2000 | 2008 | 0.3 (-0.5, 1.4) | -0.6 (-0.9, -0.4)* |
|  | 2 | 2008 | 2014 | -4.2 (-6.9, -3.1)* |  |
|  | 3 | 2014 | 2018 | 6.3 (3.8, 9.9)* |  |
|  | 4 | 2018 | 2023 | -3.2 (-5.3, -1.6)* |  |
| Mississippi | 1 | 2000 | 2008 | -1.1 (-4.6, 0.2) | -0.9 (-1.2, -0.6)* |
|  | 2 | 2008 | 2014 | 2.4 (0.0, 6.3)* |  |
|  | 3 | 2014 | 2018 | -5.7 (-8.8, 1.2) |  |
|  | 4 | 2018 | 2023 | -0.5 (-2.7, 4.8) |  |
| North Carolina | 1 | 2000 | 2008 | 6.9 (5.2, 9.3)* | -3.2 (-3.7, -2.7)* |
|  | 2 | 2008 | 2011 | -23.0 (-25.6, -15.1)* |  |
|  | 3 | 2011 | 2023 | -4.1 (-5.0, -2.9)* |  |
| Oklahoma | 1 | 2000 | 2002 | 29.2 (18.5, 36.6)* | 1.2 (0.8, 1.6)* |
|  | 2 | 2002 | 2008 | 0.4 (-1.0, 2.7) |  |
|  | 3 | 2008 | 2011 | -5.8 (-7.5, -2.2)* |  |
|  | 4 | 2011 | 2023 | -0.8 (-1.3, 0.6) |  |
| South Carolina | 1 | 2000 | 2013 | -4.3 (-4.6, -4.1)* | -3.2 (-3.3, -3.0)* |
|  | 2 | 2013 | 2023 | -1.6 (-2.0, -1.2)* |  |
| Tennessee | 1 | 2000 | 2010 | 5.8 (3.4, 10.1)* | 0.5 (-0.4, 1.3) |
|  | 2 | 2010 | 2023 | -3.5 (-5.8, -1.9)* |  |
| Texas | 1 | 2000 | 2008 | -1.8 (-7.0, 0.6) | -0.2 (-1.2, 0.4) |
|  | 2 | 2008 | 2011 | 19.6 (6.3, 26.1)* |  |
|  | 3 | 2011 | 2023 | -3.7 (-6.2, -2.1)* |  |
| Virginia | 1 | 2000 | 2013 | -0.7 (-1.0, -0.5)* | 0.4 (0.3, 0.6)* |
|  | 2 | 2013 | 2016 | 8.7 (5.4, 9.8)* |  |
|  | 3 | 2016 | 2023 | -0.8 (-1.7, -0.2)* |  |
| West Virginia | 1 | 2000 | 2005 | 3.1 (1.3, 8.1)* | 0.9 (0.4, 1.3)* |
|  | 2 | 2005 | 2008 | -4.2 (-5.9, -0.3)* |  |
|  | 3 | 2008 | 2021 | 0.2 (-2.2, 1.6) |  |
|  | 4 | 2021 | 2023 | 8.0 (0.6, 11.5)* |  |
| **Midwest** | 1 | 2000 | 2011 | -0.7 (-1.0, -0.4)* | 0.9 (0.8, 1.1)* |
|  | 2 | 2011 | 2014 | 4.9 (2.8, 5.8)* |  |
|  | 3 | 2014 | 2020 | -0.3 (-1.8, 0.3) |  |
|  | 4 | 2020 | 2023 | 5.3 (3.4, 8.3)* |  |
| Illinois | 1 | 2000 | 2018 | -2.3 (-2.6, -2.1)* | -1.6 (-1.8, -1.4)* |
|  | 2 | 2018 | 2023 | 0.8 (-0.5, 3.4) |  |
| Indiana | 1 | 2000 | 2008 | 1.0 (0.5, 1.7)* | -0.6 (-0.7, -0.4)* |
|  | 2 | 2008 | 2012 | -4.0 (-5.7, -2.6)* |  |
|  | 3 | 2012 | 2023 | -0.5 (-0.8, -0.0)* |  |
| Iowa | 1 | 2000 | 2010 | 1.6 (1.1, 2.3)* | 0.4 (0.2, 0.6)* |
|  | 2 | 2010 | 2014 | -3.5 (-5.7, -1.5)* |  |
|  | 3 | 2014 | 2023 | 0.8 (0.2, 2.1)* |  |
| Kansas | 1 | 2000 | 2009 | 2.2 (1.0, 4.4)* | 1.8 (1.4, 2.4)* |
|  | 2 | 2009 | 2016 | -3.4 (-9.8, -1.5)* |  |
|  | 3 | 2016 | 2023 | 7.0 (4.7, 10.8)* |  |
| Michigan | 1 | 2000 | 2010 | 0.2 (-2.1, 2.1) | 6.0 (5.2, 6.7)* |
|  | 2 | 2010 | 2013 | 41.1 (24.3, 47.9)* |  |
|  | 3 | 2013 | 2023 | 2.9 (0.5, 4.7)* |  |
| Minnesota | 1 | 2000 | 2010 | 0.7 (0.1, 1.1)* | 1.0 (0.7, 1.2)* |
|  | 2 | 2010 | 2013 | 7.4 (4.7, 8.6)* |  |
|  | 3 | 2013 | 2018 | 1.6 (0.1, 3.1)* |  |
|  | 4 | 2018 | 2021 | -7.5 (-8.9, -5.2)* |  |
|  | 5 | 2021 | 2023 | 4.6 (0.7, 8.0)* |  |
| Missouri | 1 | 2000 | 2009 | -2.8 (-5.4, -1.6)* | -1.2 (-1.6, -1.0)* |
|  | 2 | 2009 | 2021 | -1.0 (-3.2, -0.2)* |  |
|  | 3 | 2021 | 2023 | 5.0 (-0.7, 8.1) |  |
| Nebraska | 1 | 2000 | 2015 | -1.9 (-2.4, -1.1)* | -1.1 (-1.6, -0.7)* |
|  | 2 | 2015 | 2019 | -7.2 (-11.4, -3.8)* |  |
|  | 3 | 2019 | 2023 | 8.6 (4.6, 17.9)* |  |
| North Dakota | 1 | 2000 | 2023 | 0.8 (0.4, 1.3)* | 0.8 (0.4, 1.3)* |
| Ohio | 1 | 2000 | 2008 | -0.1 (-1.2, 3.7) | 1.1 (0.7, 1.5)* |
|  | 2 | 2008 | 2019 | -2.9 (-4.8, -2.3)* |  |
|  | 3 | 2019 | 2023 | 15.8 (11.2, 19.6)* |  |
| South Dakota | 1 | 2000 | 2010 | 0.9 (-0.0, 2.2) | -0.9 (-1.5, -0.5)* |
|  | 2 | 2010 | 2015 | -4.7 (-9.3, -2.4)* |  |
|  | 3 | 2015 | 2018 | 6.4 (1.5, 10.1)* |  |
|  | 4 | 2018 | 2021 | -11.7 (-14.7, -7.4)* |  |
|  | 5 | 2021 | 2023 | 6.2 (-1.6, 12.7) |  |
| Wisconsin | 1 | 2000 | 2011 | -3.4 (-4.1, -2.7)* | -1.0 (-1.3, -0.7)* |
|  | 2 | 2011 | 2015 | 5.6 (-3.6, 9.4) |  |
|  | 3 | 2015 | 2018 | -4.7 (-6.9, 4.3) |  |
|  | 4 | 2018 | 2023 | 1.5 (-0.7, 5.7) |  |
| **Northeast** | 1 | 2000 | 2004 | -0.4 (-2.9, 0.8) | 0.5 (0.3, 0.7)* |
|  | 2 | 2004 | 2009 | 1.7 (-2.0, 3.5) |  |
|  | 3 | 2009 | 2015 | -2.3 (-3.8, 3.0) |  |
|  | 4 | 2015 | 2018 | 6.0 (2.1, 7.1)* |  |
|  | 5 | 2018 | 2023 | 0.3 (-1.2, 1.1) |  |
| Connecticut | 1 | 2000 | 2007 | -3.0 (-6.1, -0.7)* | 2.8 (2.1, 3.6)* |
|  | 2 | 2007 | 2010 | 31.7 (17.9, 38.0)* |  |
|  | 3 | 2010 | 2018 | -3.5 (-10.0, -1.7)* |  |
|  | 4 | 2018 | 2023 | 6.3 (1.9, 16.7)* |  |
| Maine | 1 | 2000 | 2003 | 2.5 (1.0, 5.7)* | -1.8 (-1.9, -1.6)* |
|  | 2 | 2003 | 2007 | -1.8 (-3.3, -0.5)* |  |
|  | 3 | 2007 | 2010 | -10.0 (-11.0, -8.1)* |  |
|  | 4 | 2010 | 2013 | 3.8 (1.7, 4.8)* |  |
|  | 5 | 2013 | 2023 | -2.1 (-2.5, -1.8)* |  |
| Massachusetts | 1 | 2000 | 2016 | -1.1 (-1.8, -0.6)* | 0.6 (0.2, 1.0)* |
|  | 2 | 2016 | 2023 | 4.8 (3.0, 7.9)* |  |
| New Hampshire | 1 | 2000 | 2002 | 7.7 (4.2, 11.5)* | -0.8 (-1.0, -0.6)* |
|  | 2 | 2002 | 2005 | -7.7 (-9.3, -5.8)* |  |
|  | 3 | 2005 | 2008 | 5.6 (3.1, 7.3)* |  |
|  | 4 | 2008 | 2014 | -4.1 (-6.1, -3.4)* |  |
|  | 5 | 2014 | 2023 | -0.1 (-0.6, 0.6) |  |
| New Jersey | 1 | 2000 | 2003 | 3.2 (-4.0, 9.1) | 0.5 (0.0, 0.9)* |
|  | 2 | 2003 | 2006 | 15.1 (-2.9, 18.2) |  |
|  | 3 | 2006 | 2023 | -2.4 (-2.9, -1.8)* |  |
| New York | 1 | 2000 | 2005 | -0.8 (-4.0, 0.7) | -0.7 (-1.0, -0.4)* |
|  | 2 | 2005 | 2009 | 2.1 (-5.6, 4.4) |  |
|  | 3 | 2009 | 2014 | -5.6 (-8.5, 4.9) |  |
|  | 4 | 2014 | 2017 | 4.9 (-2.3, 6.8) |  |
|  | 5 | 2017 | 2023 | -0.9 (-2.9, 1.1) |  |
| Pennsylvania | 1 | 2000 | 2009 | 2.7 (1.4, 6.2)* | 4.4 (3.9, 5.0)* |
|  | 2 | 2009 | 2015 | -3.5 (-8.8, -1.3)* |  |
|  | 3 | 2015 | 2018 | 54.3 (40.6, 61.6)* |  |
|  | 4 | 2018 | 2023 | -6.4 (-10.5, -3.5)* |  |
| Rhode Island | 1 | 2000 | 2008 | -2.3 (-2.7, -1.6)* | -1.6 (-1.7, -1.4)* |
|  | 2 | 2008 | 2011 | -8.0 (-8.9, -5.3)* |  |
|  | 3 | 2011 | 2023 | 0.6 (0.3, 1.0)* |  |
| Vermont | 1 | 2000 | 2008 | 1.5 (1.0, 2.1)* | 0.4 (0.2, 0.6)* |
|  | 2 | 2008 | 2011 | -7.7 (-9.1, -5.2)* |  |
|  | 3 | 2011 | 2015 | 4.1 (2.6, 6.9)* |  |
|  | 4 | 2015 | 2020 | -2.7 (-5.1, -1.5)* |  |
|  | 5 | 2020 | 2023 | 6.9 (4.2, 11.8)* |  |

Abbreviations: AAPC = average annual percent change, APC = annual percent change.

Asterisk (*) indicates AAPC/APC is statistically different from 0.0 at the 0.05 level.
